# Lack of direct cytotoxicity of extracellular ATP against hepatocytes: role in the mechanism of acetaminophen hepatotoxicity

**DOI:** 10.18053/jctres.201502.004

**Published:** 2015-09-30

**Authors:** Yuchao Xie, Benjamin L. Woolbright, Milan Kos, Mitchell R. McGill, Kenneth Dorko, Sean C. Kumer, Timothy M. Schmitt, Hartmut Jaeschke

**Affiliations:** 1 Department of Pharmacology, Toxicology and Therapeutics, University of Kansas Medical Center, Kansas City, United States; 2 Department of Experimental Surgery, Academic Medical Center, University of Amsterdam, Amsterdam, The Netherlands; 3 Department of Surgery, University of Kansas Medical Center, Kansas City, United States

**Keywords:** Acetaminophen hepatotoxicity, damage-associated molecular patterns, sterile inflammation, necrosis, human hepatocytes

## Abstract

**Background:**

Acetaminophen (APAP) hepatotoxicity is a major cause of acute liver failure in many countries. Mechanistic studies in mice and humans have implicated formation of a reactive metabolite, mitochondrial dysfunction and oxidant stress as critical events in the pathophysiology of APAP-induced liver cell death. It was recently suggested that ATP released from necrotic cells can directly cause cell death in mouse hepatocytes and in a hepatoma cell line (HepG2).

**Aim:**

To assess if ATP can directly cause cell toxicity in hepatocytes and evaluate their relevance in the human system.

**Methods:**

Primary mouse hepatocytes, human HepG2 cells, the metabolically competent human HepaRG cell line and freshly isolated primary human hepatocytes were exposed to 10-100 µM ATP or ATγPin the presence or absence of 5-10 mM APAP for 9-24 h.

**Results:**

ATP or ATγP was unable to directly cause cell toxicity in all 4 types of hepatocytes. In addition, ATP did not enhance APAP-induced cell death observed in primary mouse or human hepatocytes, or in HepaRG cells as measured by LDH release and by propidium iodide staining in primary mouse hepatocytes. Furthermore, addition of ATP did not cause mitochondrial dysfunction or enhance APAP-induced mitochondrial dysfunction in primary murine hepatocytes, although ATP did cause cell death in murine RAW macrophages.

**Conclusions:**

It is unlikely that ATP released from necrotic cells can significantly affect cell death in human or mouse liver during APAP hepatotoxicity.

**Relevance for patients:**

Understanding the mechanisms of APAP-induced cell injury is critical for identifying novel therapeutic targets to prevent liver injury and acute liver failure in APAP overdose patients.

## 1. Introduction

Acetaminophen, a safe analgesic drug at therapeutic doses, can induce severe liver injury and even liver failure after overdose in experimental animals and in humans [1,2]. Although the focus of pathophysiological studies during the last four decades was on intracellular signaling mechanisms [3-5], in recent years a contribution of the innate immune response has been discussed [6]. A sterile inflammatory response is activated by release of damage-associated molecular patterns (DAMPs) such as nuclear DNA fragments, mitochondrial DNA, high mobility group box 1 protein (HMGB1) and ATP from necrotic hepatocytes [2,7-9]. The activation of toll-like receptors on macrophages by DAMPs leads to the transcriptional activation of pro-inflammatory cytokines including pro-IL-1β. In addition, stimulation of purinergic receptors by ATP activates the Nalp3 inflammasome, triggering the activation of caspase-1 which processes pro-IL-1β to the active cytokine [10]. Although we could not confirm a role of caspase-1, the Nalp3 inflammasome and IL-1β [11, 12], or the purinergic receptor P2X7 [13] in the mechanism of APAP hepatotoxicity, a recent paper introduced a novel hypothesis for the role of ATP released by necrotic cells. Amaral et al. [14] provided evidence that human hepatoma cells (HepG2) exposed to APAP release ATP into the culture supernatant. In addition, the authors further demonstrated that primary mouse hepatocytes and HepG2 cells can be directly killed by exposure to ATP at concentrations between 10 and 100 µM [14]. Thus, in addition to activating the inflammasome, ATP may directly contribute to aggravation of APAP-induced liver injury as a cytotoxic agent [14]. This novel concept would support a previous hypothesis that “death factors” released by necrotic cells are involved in the expansion of the original injury [15]. If this hypothesis can be supported, any ATP leaking out of necrotic cells could be potentially dangerous for neighboring cells. This novel hypothesis could have a substantial impact not only on the mechanism of APAP hepatotoxicity but also on a large number of other liver disease models, e.g. ischemia-reperfusion injury, where extensive necrosis occurs [16-18]. Therefore, the objective of the current study was to determine if ATP is directly cytotoxic in 4 different cell culture models: primary cultured mouse and human hepatocytes and a metabolically competent (HepaRG) and a hepatoma cell line (HepG2), which does not express cytochrome P450 and other drug metabolizing enzymes and transporters [19]. In addition, ATP toxicity was tested in murine RAW macrophages. Alternatively, it was hypothesized that ATP could aggravate cell death during APAP hepatotoxicity.

## 2. Material and Methods

### 2.1. HepaRG and HepG2 cell lines

HepaRG cells were acquired from Biopredic International (Rennes, France).The detailed process of HepaRG cell culture was described previously [20]. HepG2 cells obtained from ATCC (Manassas, VA, USA) were cultured in DMSO (dimethyl sulfoxide)-free Williams’ E medium containing penicillin/streptomycin, insulin and 10% fetal bovine serum, and were 70%~80% confluent before treatment. Both cell lines were treated with 10 mM APAP (Sigma, St. Louis, MO, USA; Lot #36F-7005-1) dissolved in warm Williams’ E medium. ATP (Sigma) was dissolved in saline and added to the cell culture medium to a final concentration of 10 µM or 100 µM. Twenty-four hours after treatment of either ATP and/or APAP, medium and cell fractions were harvested, and cell viability was determined using the lactate dehydrogenase (LDH) assay [21].

### 2.2. Primary mouse hepatocytes

Primary mouse hepatocytes were isolated by a two-step isolation procedure as previously described in detail [21]. The animals used were 8 week old male C57Bl/6J mice (Jackson Laboratories, Bar Habor, Maine, USA).Animals were acclimatized for at least 3 days and fasted overnight before cell isolation. All experimental protocols were approved by the Institutional Animal Care and Use Committee of the University of Kansas Medical Center. Generally, cell viability of each isolation was more than 90%, and cell purity was >95% hepatocytes. Cells were treated with 5 mM APAP, which was dissolved in warm Williams’ E medium. ATP or ATγPwere dissolved in saline and added to the cell culture medium to a final concentration of 10 µM, 100 µM, 1 mM or 10 mM. All cells and supernatants were harvested 9 h or 24 h after the treatment. The LDH assay was carried out to assess the cell death.In addition, propidium iodide staining and JC-1 fluorescence assays were performed as described previously [20, 22].

### 2.3. Primary human hepatocytes

Freshly isolated primary human hepatocytes were obtained from the Human Cell Isolation Core in the Department of Pharmacology, Toxicology and Therapeutics at The University of Kansas Medical Center. The liver sources were either unused tissue from donor livers or material from liver resections. All human tissues were obtained with informed consent according to ethical and institutional guidelines. Hepatocytes were isolated as previously described in detail [22] using a 3 step process and the dissociation enzymes collagenase (Vitacyte, Indianapolis, Indiana, USA) and protease (Vitacyte). Human hepatocytes were plated in Hepatocyte Maintenance Medium (HMM, Lonza Group, Basel, Switzerland) supplemented with dexamethasone, insulin and gentamicin/amphotericin-B (Lonza) and 5% newborn calf serum (Atlanta Biologicals, Lawrenceville, Georgia, USA) was used to maintain the hepatocytes. The hepatocytes were allowed to attach for 2 hours in a 37°C, 5% CO_2_ incubator before treatment. Cells were treated with 10 mM APAP dissolved in warm HMM, and all cells were harvested at 24 h post-treatment. For ATP treatment, ATP was dissolved in saline and added to the cell culture medium to a final concentration of 10 µM or 100 µM. After cell harvesting, alanine aminotransferase (ALT) activities were measured in cells and the supernatant using an ALT reagent kit (Pointe Scientific, Michigan, USA).

### 2.4. Macrophage Studies

The RAW264.7 NF-κB/LUCPorter™ (Novus Biologicals, Littleton, CO) cell line used in this study is a stably transfected RAW264.7 murine macrophage cell line that expresses an optimized Renilla luciferase reporter gene (RenSP) under the transcriptional control of an NF-κB response element. The cells were a kind gift from Prof. dr. Menno P.J. de Winther, Department of Medical Biochemistry, Academic Medical Center, University of Amsterdam, The Netherlands. RAW264.7 cells were grown in T75 flasks at standard culture conditions (37°C, 5% CO_2_, and 95% air) and cultured in Dulbecco’s Modified Eagle Medium (DMEM) (Lonza Biowhittaker®, Verviers, Belgium) culture medium supplemented with 10% fetal bovine serum (FBS) (v/v) (Bodinco, Alkmaar, The Neth-erlands), 1% penicillin/streptomycin (v/v), and 1% L-glutamine (v/v) (both from Lonza, Walkersville, MD). Cells were passaged twice a week at a 1:8 or 1:16 ratio following harvesting with a cell scraper (Corning Inc., Corning, NY) and received fresh medium every 2-3 days. RAW264.7 cells were seeded in 24-wells plates at a density of 2.0 × 10^5^ or 2.5 × 10^4^ cells/well and cultured for 24 h before being incubated with increasing concentrations ATP (Sigma Aldrich, St. Louis, USA) or vehicle (0.9 % NaCl) in 0.5 mL fully supplemented DMEM (negative control) at standard culture conditions for 9 hours or 72 hours, respectively. Cell viability was determined based on the measurement of cellular protein content using the sulforhodamine B (SRB) colorimetric assay [23].

### 2.5. Statistics

All results were expressed as mean ± SE. Comparisons between multiple groups were performed with the Kruskal-Wallis Test (nonparametric ANOVA) followed by Dunn’s Multiple Comparisons Test. P < 0.05 was considered significant.

## 3. Results

### 3.1. Primary mouse hepatocytes

**Figure 1.**
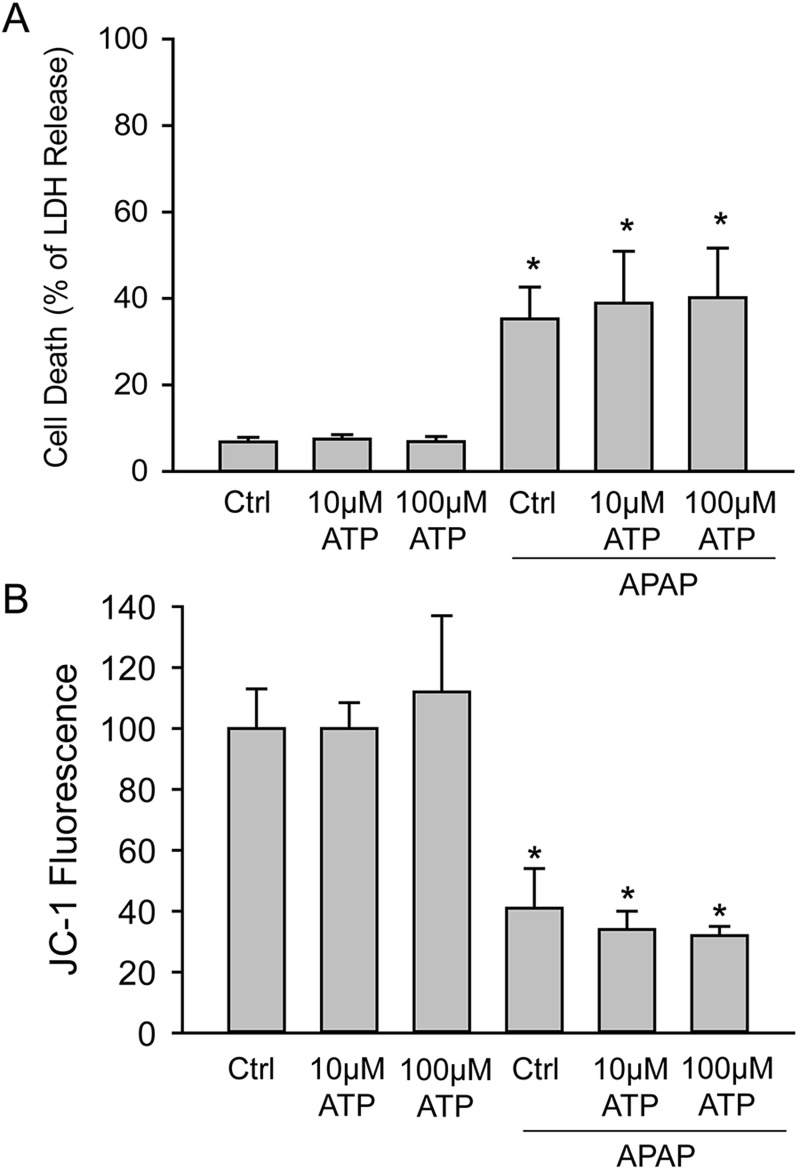
Effect of ATP on primary mouse hepatocytes. Primary mouse hepatocytes were treated with 5 mM acetaminophen dissolved in Williams’ E medium, and cell death was assessed by the percentage of lactate dehydrogenase (LDH) released into the medium from the cells at 9 h after the treatment (A). ATP was freshly dissolved in saline and added to the cell culture medium to a final concentration of 10 µM or 100 µM at the time of APAP treatment. JC-1 fluorescence was measured to assess mitochondrial dysfunction (B). Data represent mean ± SE of *n*=4 different mouse hepatocytes isolations *p<0.05 (compared to corresponding groups without APAP).

**Figure 2.**
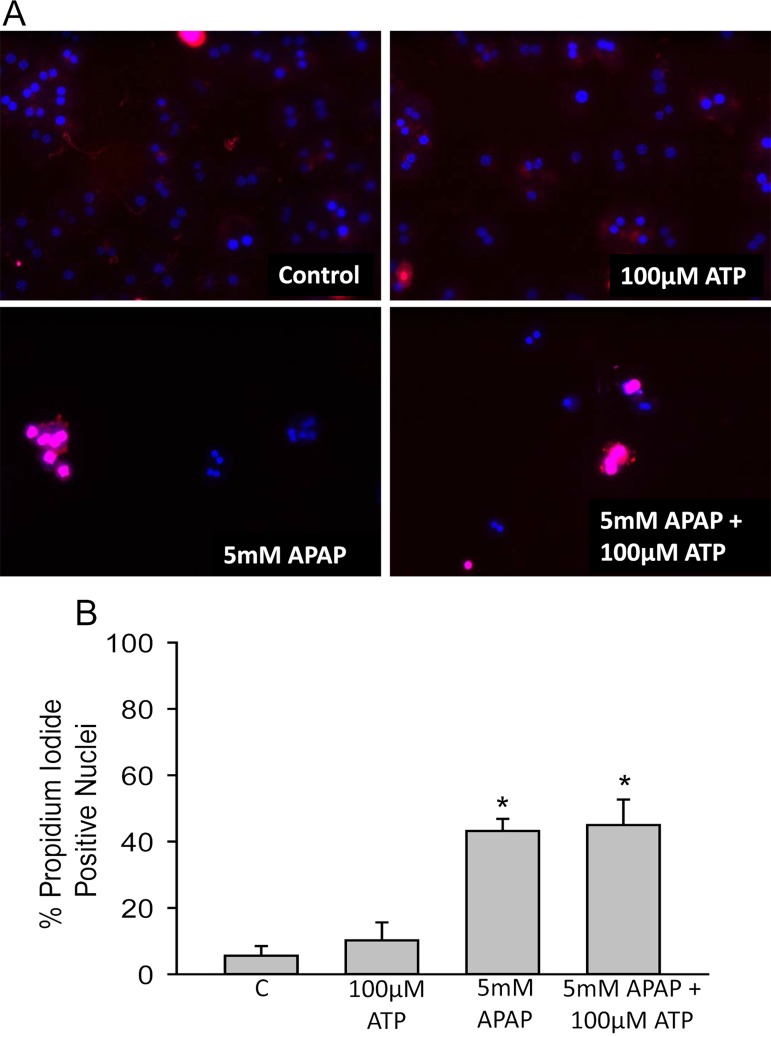
ATP does not increase cell death in murine hepatocytes exposed to APAP. Primary mouse hepatocytes were treated with ATP in the presence or absence of 5 mM acetaminophen and cell death was assessed by propidium iodide/DAPI staining (A). Quantification was done by counting PI-positive cells in four different fields per well from different cell isolations. Data represent mean ± SE of *n* = 3 different mouse hepatocytes isolations *p<0.05 (compared to corresponding groups without APAP).

**Figure 3.**
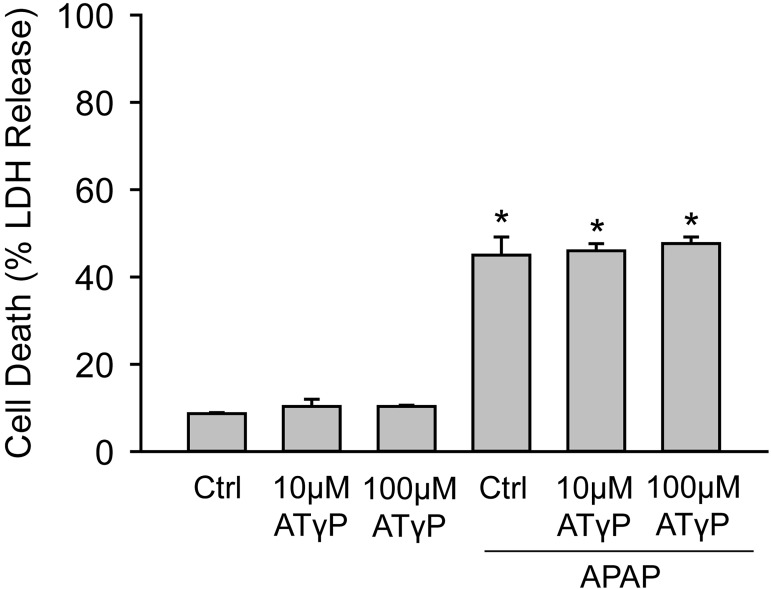
Non-hydrolyzable ATP (ATγP) does not increase cell death in hepatocytes. Primary mouse hepatocytes were treated with 10 µM or 100 µM ATγP in the presence or absence of 5 mM acetaminophen and cell death was assessed by LDH release. Data represent mean ± SE of *n* = 3 different mouse hepatocytes isolations *p<0.05 (compared to corresponding groups without APAP).

Previous results showed that cultured hepatocytes release ATP into the supernatant after exposure to 20 mM APAP resulting in concentrations of up to 10 µM [14]. Therefore, primary mouse hepatocytes were exposed to 10 or 100 µM ATP in the presence or absence of 5 mM APAP for 9 h (Figure 1). Based on LDH release as indicator for cell necrosis [21], ATP alone did not cause more cell death compared to untreated mouse hepatocytes. In addition, ATP did not trigger additional necrosis in cells exposed to APAP (Figure 1A). Similar results were obtained when JC-1 fluorescence was measured as indicator of the mitochondrial membrane potential (Figure 1B). ATP alone did not cause mitochondrial dysfunction and ATP did not aggravate APAP-induced loss of the mitochondrial membrane potential (Figure 1B). These results were similar to experiments with ATP concentrations of up to 10 mM and longer time points (24 h) (data not shown). To confirm these results, propidium iodide staining was performed to assess the number of necrotic cells (Figure 2). No differences were observed between the ATP and control groups or the APAP and APAP + ATP groups; the overall cell death was consistent with previous results. Finally, a non-hydrolyzable form of ATP (ATγP) was used in place of ATP as described by Amaral et al. (2013) and LDH was measured to assess cell death (Figure 3). The use of the ATγP yielded the same results, as no increase in cell death was found with ATγP alone or with co-treatment of ATγP and APAP (Figure 3).

### 3.2. Primary human hepatocytes and hepatoma lines

**Figure 4.**
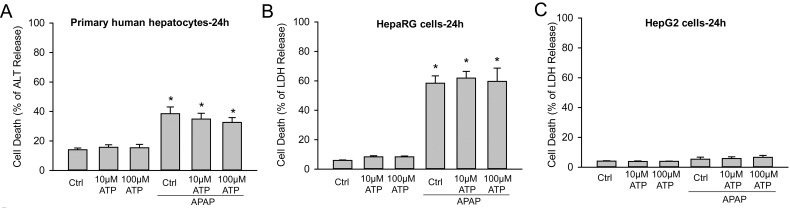
ATP does not increase cell death in human hepatocytes or hepatoma cell lines. Primary human hepatocytes (A), HepaRG cells (B) or HepG2 cells were treated with ATP in the presence or absence of 5 mM acetaminophen and cell death was assessed by ALT or LDH release. Data represent mean ± SE of n=3 different HepaRG cell differentiations, n=3 HepG2 cell preparations and n=8 human hepatocyte isolations. * *p* <0.05 (compared to corresponding groups without APAP).

Using freshly isolated primary human hepatocytes, a moderate degree of necrosis (ALT release) was observed after exposure to 10 mM APAP for 24 h as compared to untreated cells (controls) (Figure 4A). Similar to primary mouse hepatocytes, exposure to ATP did not cause any additional cell death in human hepatocytes compared to untreated cells or compared to APAP-treated cells (Figure 4A). The same results were obtained in the metabolically competent HepaRG cell line cultured with the same concentrations of APAP and ATP; again ATP failed to increase cell death in any treatment (Figure 4B). Because previous studies showed evidence of ATP cytotoxicity in HepG2 cells [14], HepG2 cells were exposed to ATP in the presence or absence of 10 mM APAP. Similar to our previous findings [20], APAP did not cause cell death in HepG2 cells (Figure 4C). More importantly, neither 10 nor 100 µM ATP caused cell death in control HepG2 cells or in cells exposed to APAP (Figure 4C).

### 3.3. Cytotoxicity of ATP in the macrophage cell line RAW264.7

**Figure 5.**
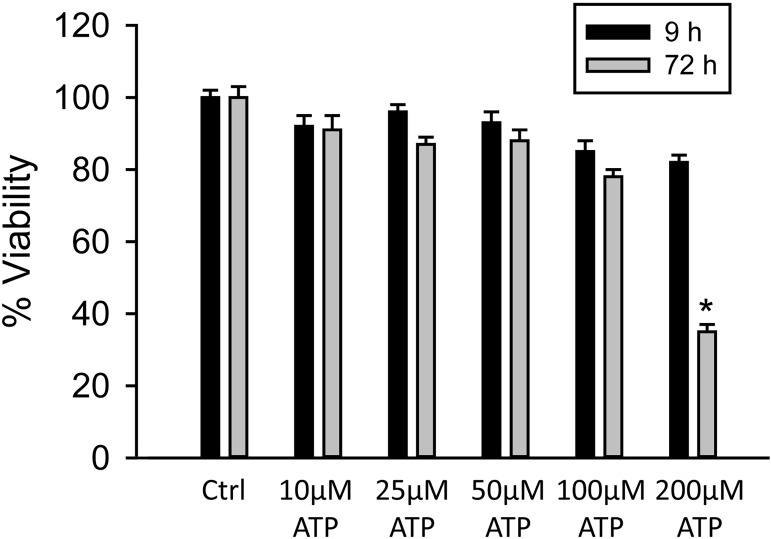
Cytotoxicity of ATP in RAW 264.7 macrophages. RAW macrophages were exposed to increasing concentrations of ATP for 9 or 72 h, respectively, and then cell viability was measured via the SRB test. Data represent mean SE of n=4 different experiments. *p<0.05 (compared to Ctrl).

We measured ATP based-cytotoxicity in a macrophage cell line that expressed purinergic receptors [24]. Stimulating macrophages with ATP at concentrations up to 200 µM produced no toxicity with an incubation time of 9 h; however, longer incubation times (72 h) produced significant toxicity at a concentration of 200 µM (Figure 5). While these concentrations are extremely high and certainly well beyond even pathophysiological concentrations, these data do indicate that ATP is both stable and active *in vitro*, and can produce toxicity given long incubation times and higher concentrations. These data are supported by previous studies on the biological activity and the stability of ATP using a hexokinase assay [25].

## 4. Discussion

The objective of this investigation was to determine if ATP can act as a direct cytotoxic agent against hepatocytes or can aggravate APAP-induced cell death. This hypothesis was tested in 4 different types of hepatocytes, i.e., primary mouse hepatocytes, primary human hepatocytes, a metabolically competent human hepatoma cell line (HepaRG) and a human hepatoma cell line that does not express CYPs or other drug metabolizing enzymes (HepG2). Consistent in all 4 cell types, various doses of ATP were unable to directly cause cell death and were also not able to enhance APAP-induced cell necrosis; ATγP, a non-hydrolyzable form of ATP, was equally ineffective to increase cell death in murine hepatocytes. In contrast to the different types of hepatocytes, ATP caused cell death in macrophages at high concentrations.

### 4.1. ATP as activator of the inflammasome

The initial hypothesis was that ATP released during necrotic cell death can activate the Nalp3 inflammasome and caspase-1 through binding to the P2X7 receptor and thereby stimulate the processing of IL-1β, which then promotes neutrophil infiltration into the liver and an aggravation of liver injury by these inflammatory cells [10]. Although an activation of the Nalp3 inflammasome and a caspase-1-dependent processing of pro-IL1β could be confirmed [11], there was no effect of endogenous IL-1β on the inflammatory response and liver injury after APAP overdose [11]. In addition, treatment with very high doses of exogenous IL-1β or endotoxin had no effect on APAP-induced liver injury [11, 26]. Furthermore, IL-1 receptor-deficient mice showed similar injury as wild type animals [11] as did various KO mice of the Nalp3 inflammasome components [12], animals deficient of various adhesion molecules [26, 27] or NADPH oxidase-deficient mice [28, 29]. Most importantly, the P2X7 inhibitor used by Hoque et al.[10] was shown to inhibit P450 enzymes and thus protects because it prevents the initiation of injury rather than the activation of the inflammasome [13]. Together, these data strongly argue against a relevant contribution of the ATP-activated inflammasome to the injury after APAP overdose. Consistent with these *in vivo* animal data, neutrophil activation in human APAP overdose patients occurs only during regeneration but not during the active injury phase [29] where most DAMPs including ATP are being released [2,7,8,30]. In addition, there is virtually no IL-1β formation in humans during APAP hepatotoxicity [31] suggesting that also in humans the Nalp3 inflammasome is of limited relevance.

### 4.2. ATP as direct cytotoxic agent

Recently, a second hypothesis was introduced. Amaral et al. [14] reported that HepG2 cells release ATP after exposure to 20 mM APAP for 0.5-2 hours. The peak levels of ATP measured were approximately 10 µM at 0.5 hours [14]. Using 10 µM and 100 µM ATP, the authors then demonstrated that these doses caused 40% cell death in primary mouse hepatocytes isolated from C57Bl/6J mice and in HepG2 cells after 18-24 h exposure [14]. Based on these findings, the authors concluded that ATP, in addition to activating the inflammasome through purinergic receptors on macrophages, can also act as a direct cytotoxic agent against hepatocytes [14]. This cytotoxic effect was mediated through purinergic receptors, which are known to be expressed on hepatocytes [32, 33]. However, in our hands, ATP in concentrations from 10 µM up to 10 mM did not affect cell viability in primary mouse hepatocytes isolated from the same strain of mice, nor did treatment with a non-hydrolyzable ATP analogue (ATγP). Moreover, 5 mM APAP caused significant cell death at 9 h post-treatment in our primary mouse hepatocytes, but various concentrations of ATP or ATγP had no significant impact on this APAP-induced cell death. Cell death in these experiments as indicated by LDH or ALT release was confirmed by additional parameters such as propidium iodide staining, as measure of necrosis, and by the JC-1 assay, which indicates the mitochondrial membrane potential. Thus, ATP at levels that would be present in the extracellular milieu *in vivo* for only a short period of time and at concentrations which are one to several orders of magnitude beyond physiological levels could not kill mouse hepatocytes over a 24 h exposure time. Furthermore, there was no evidence that the same physiological or supraphysiological concentrations of ATP or ATγP could enhance APAP-induced cell death. Based on these findings, we have to conclude that ATP is not a direct cytotoxic agent during APAP-induced cell death of cultured murine hepatocytes. In contrast, some cytotoxicity was observed in murine macrophages exposed to supraphysiological concentrations of ATP confirming the idea that ATP could potentially be toxic *in vitro*, but only under circumstances largely irrelevant to human biology.

Additional experiments with different hepatocyte cell types confirmed these findings. First, the metabolically competent human hepatocyte cell line HepaRG [34], which is sensitive to APAP toxicity [20], could not be killed by ATP alone, and ATP could not enhance APAP-induced cell death in these cells. Similarly, the viability of HepG2 cells, which are known to have very low drug metabolizing enzyme levels [19] and are thus mostly resistant to APAP-induced necrosis [20], was not affected by ATP alone or in combination with APAP. Finally, primary human hepatocytes, which were sensitive to APAP [22], did not show loss of cell viability with ATP alone and did not show more cell death when ATP was combined with APAP.

In summary, our data consistently demonstrated in 4 different types of hepatocytes (human and mouse) that extracellular ATP was unable to directly cause cell death or aggravate APAP-induced cell death. Thus, it is unlikely that ATP released from necrotic cells can significantly affect cell death in the liver during APAP hepatotoxicity or other pathophysiologies that are characterized by extensive necrosis. APAP-induced cell death is clearly dominated by intracellular events caused by reactive metabolite formation, mitochondrial dysfunction and oxidant stress rather than extracellular mediators such as ATP. However, DAMP-induced cytokine formation *in vivo* can affect intracellular signaling events and enhance cell death [35] or recruit inflammatory cells in preparation for regeneration [6].
